# Experimental data on synthesis and characterization of chiral dinuclear manganese (II-II) compounds as biomimetic models of the active center of catalase

**DOI:** 10.1016/j.dib.2019.104883

**Published:** 2019-12-02

**Authors:** Yenny Ávila-Torres, Jorge Acosta, Lázaro Huerta, Alfredo Toscano, Felipe J. González, Norah Barba Behrens

**Affiliations:** a Grupo de Investigación QUIBIO, Facultad de Ciencias Básicas, Universidad Santiago de Cali, Santiago de Cali, Pampalinda, Colombia; b Instituto de Investigaciones en Materiales, Universidad Nacional Autónoma de México, C.U., Coyoacán, México D.F., 04510, Mexico; c Instituto de Química, Universidad Nacional Autónoma de México, C.U., Coyoacán, México D.F., 04510, Mexico; d Departamento de Química, Cinvestav México, AP 14-740, CP 07000, México DF, Mexico

**Keywords:** Manganese-catalases, Electronic properties, Magnetic properties, Functional structure, Chiral ligands

## Abstract

Dinuclear manganese (II– III) compounds, which are potential models of the active center of catalase, were synthetized. This type of metalloenzymes presents biological importance due to three factors: they are redox catalyst centres, they are able to carry out hydrolytic reactions and they participate in activated processes via Lewis acids. Structurally, their active centre is composed by dinuclear manganese compounds, linked to nitrogen and oxygen donor atoms. An octahedral geometry around the metal ions were found, with acetate, hydroxy and aquo ligands; which can work as molecule bridges between them. The acid medium favours the electronic transfer between Mn^3+^ - Mn^2+^ as redox centre at 1.559 V and the consequent oxidation of hydrogen peroxide or organic molecules. The work also reports the data of two chiral novel compounds, [Mn_2_(*S,S(*+)Hcpse)_4_(NaClO_4_)_2_(NaOH)(CH_4_O)]_n_·[(C_2_H_6_O)_2_]_n_·[(CH_4_O)_2_]_n_ and its respective enantioisomer, in which μ-oxo being as bridge metal centre. The X-ray structural was obtained as well as the optical and magnetic properties using Circular Dichroism, Electronic Paramagnetic Resonance, Magnetic Susceptibility and X-ray photoelectron spectroscopy.

**Table:** Specifications Table

Subject	Inorganic Chemistry
Specific subject area	Bioinorganic chemistry, Science Materials
Type of data	Tables and Figures
How data were acquired	X-ray crystallographic study (DRX) Valence metal centres (XPS) Chiral activity (Circular dichroism) Electrochemical study (Voltammetry cyclic) Magnetic properties (Susceptibility magnetic at variable temperature)
Data format	Raw
Parameters for data collection	The data shown in this article are obtained from the methanol synthesis of the tridentate ligand - N acetyl pseudoephedrine with manganese (II), as structural biomimetic models of catalase and peroxidases. Likewise, its structural (X- ray crystal), optical (CD spectra), electronic (electrochemical analyses) and magnetic (EPR and susceptibility magnetic) characterization was carried out.
Description of data collection	The data collection was obtained of the synthesis and crystal structures of two manganese (II) complexes containing chiral aminoalcohols (*R,R* (−)H_2_cpse) and (*S*,*S* (+)H_2_cpse). The 1:1 reaction between Mn(CH_3_COOH)·4H_2_O and ligands, followed by the addition of NaOH and NaClO_4_ in methanol afforded the polymeric compounds. In solution, a change of the oxidation state of the manganese ion was observed using voltammetry cyclic and circular dichroism in comparison with XPS, which will serve to understand the electronic exchange in dissolution when using these models as catalase or peroxidases.
Data source location	Institution: Universidad Santiago de Cali City/Town/Region: Santiago de Cali Country: Colombia
Data accessibility	The data are found only in this article.

**Table:** 

**Value of the Data** • The new chiral ligands with manganese (II) reported are new interesting biomimetic models of metalloenzymes such as catalases. The characterization of these synthetic models emulating biological processes allows the design of new structures and to test experimentally the models reported here. • Data can be used to compare the stability of the biomimetic compounds in solid state and in solution. • Data are useful in the study of the mechanism of chiral compounds action in oxidases, which oxidize organic molecules and substrates such as hydrogen peroxide with several potential applications in water treatment and decorating dyes among others. • These data show molecules that are related to enantioselective synthesis, where the metallic centre and the chiral ligand provide important optical properties that can affect intermolecular interactions in processes associated with metalloenzymes, such as: manganese peroxidases and catalase manganese.

## 1 Data

**Fig. 1:**
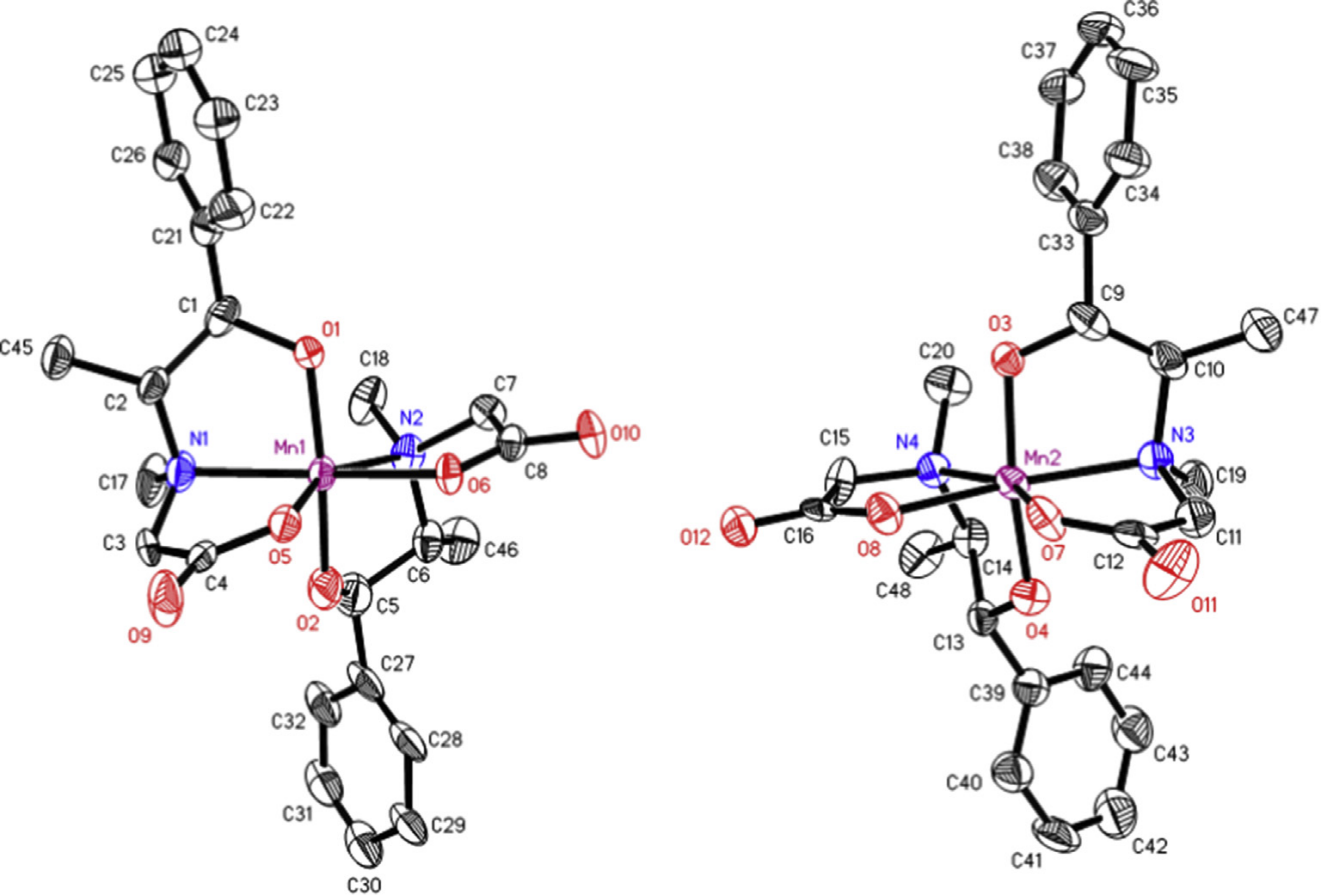
Ortep representation of the *[Mn*_*2*_*(S,S(* + *)Hcpse)*_*4*_*(NaClO*_*4*_*)*_*2*_*(NaOH)(CH*_*4*_*O)]*_*n*_*[(C*_*2*_*H*_*6*_*O)*_*2*_*]*_*n*_*·[(CH*_*4*_*O)*_*2*_*]*_*n*_ compound.

**Fig. 2:**
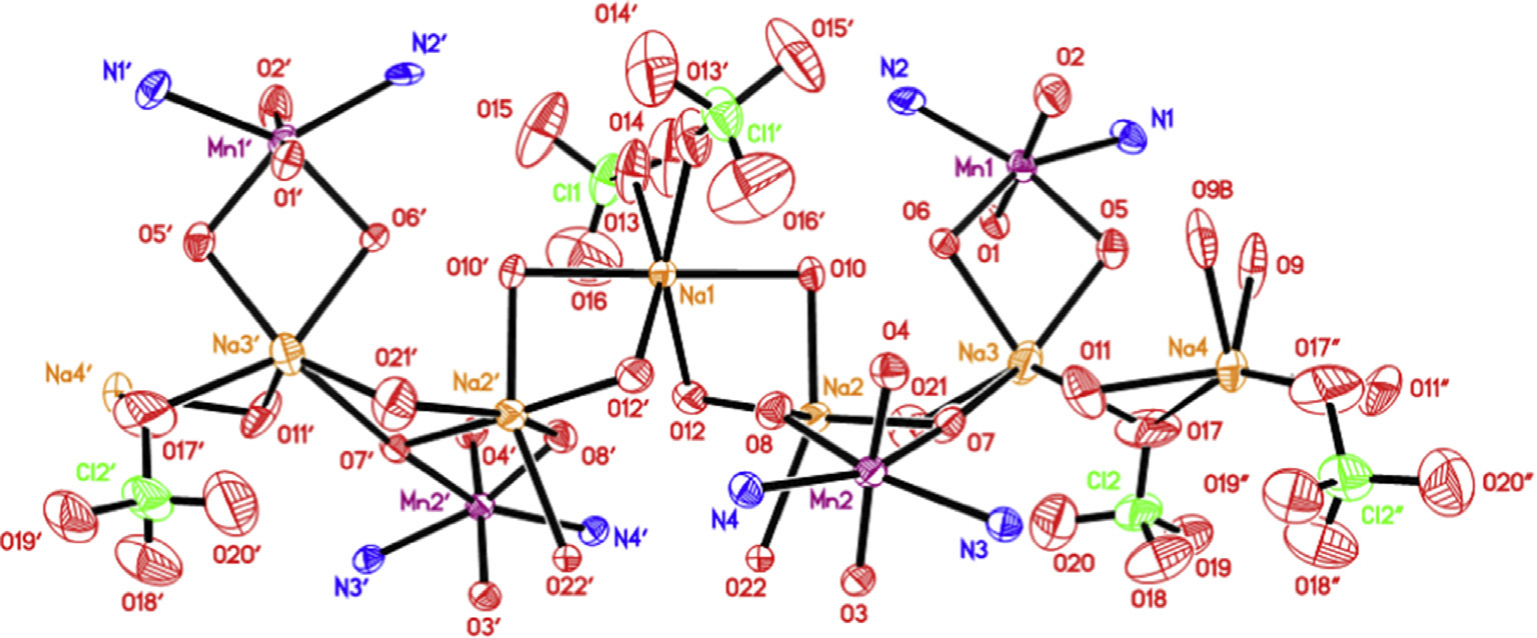
Polymeric structure for *[Mn*_*2*_*(S,S(* + *)Hcpse)*_4_*(NaClO*_4_*)*_2_*(NaOH)(CH*_4_*O)]*_*n*_*[(C*_2_*H*_6_*O)*_2_*]*_n_*·[(CH*_4_*O)*_2_*]*_n_ compound.

**Fig. 3:**
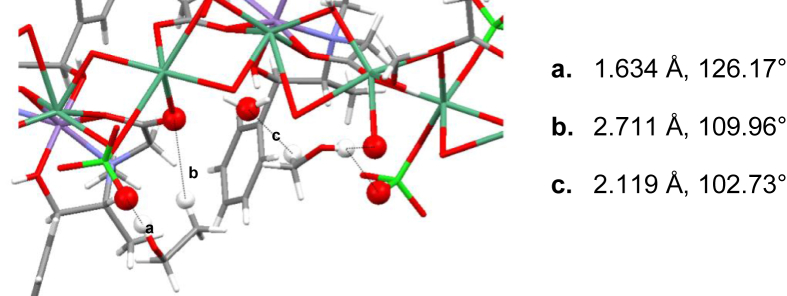
Intermolecular interactions for *[Mn*_*2*_*(S,S(* + *)Hcpse)*_*4*_*(NaClO*_*4*_*)*_*2*_*(NaOH)(CH*_*4*_*O)]*_*n*_*[(C*_*2*_*H*_*6*_*O)*_*2*_*]*_*n*_*·[(CH*_*4*_*O)*_*2*_*]*_*n*_.

**Fig. 4:**
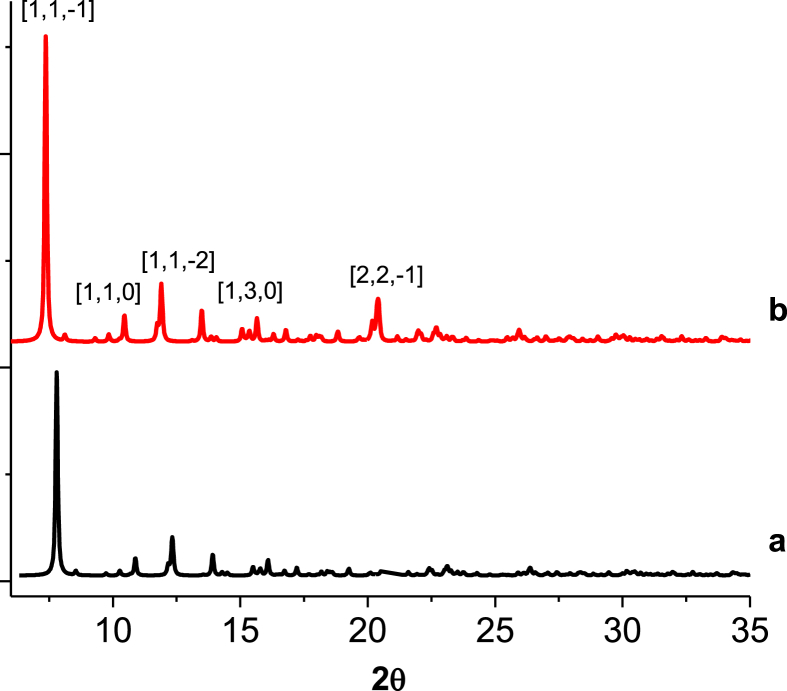
X-ray powder diffraction for: *a) [Mn*_*2*_*(S,S(* + *)Hcpse)*_*4*_*(NaClO*_*4*_*)*_*2*_*(NaOH)(CH*_*4*_*O)]*_*n*_*·[(C*_*2*_*H*_*6*_*O)*_*2*_*]*_*n*_*·[(CH*_*4*_*O)*_*2*_*]*_*n*_, *b) [Mn*_*2*_*(R,R(−)Hcpse)*_*4*_*(NaClO*_*4*_*)*_*2*_*(NaOH)(CH*_*4*_*O)]*_*n*_*·[(C*_*2*_*H*_*6*_*O)*_*2*_*]*_*n*_*·[(CH*_*4*_*O)*_*2*_*]*_*n*_.

**Fig. 5:**
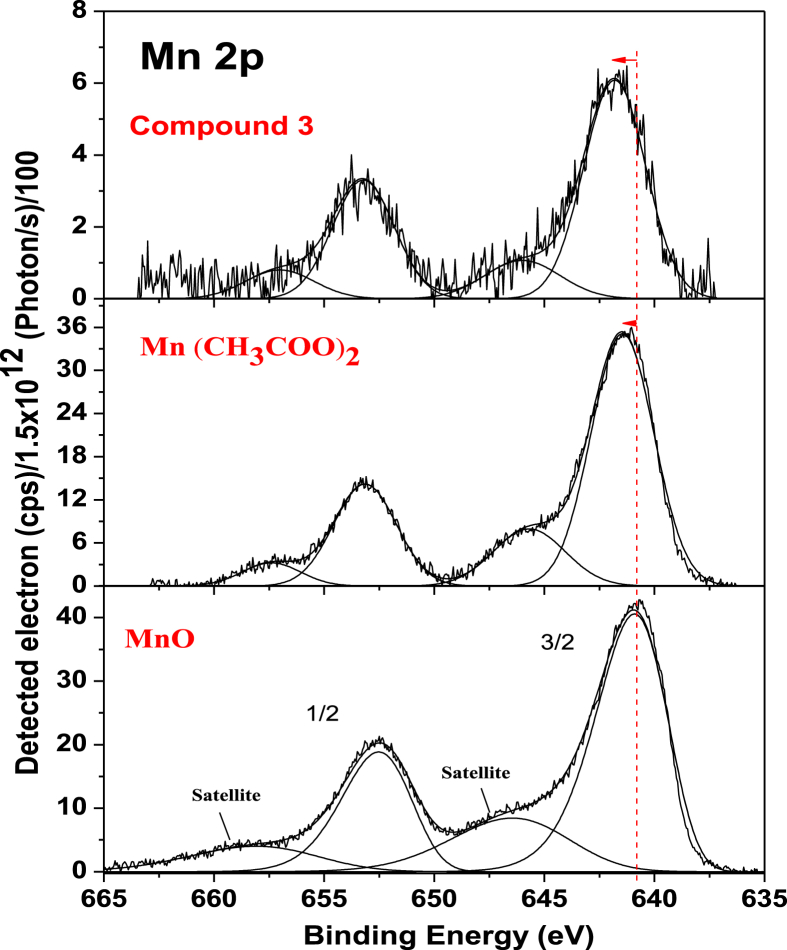
XPS spectra of Mn 2p core level of MnO, Mn(CH_*3*_COO)_*2*_ and compound *[Mn*_*2*_*(S,S(* + *)Hcpse)*_*4*_*(NaClO*_*4*_*)*_*2*_*(NaOH)(CH*_*4*_*O)]*_*n*_*·[(C*_*2*_*H*_*6*_*O)*_*2*_*]*_*n*_*·[(CH*_*4*_*O)*_*2*_*]*_*n*_.

**Fig. 6:**
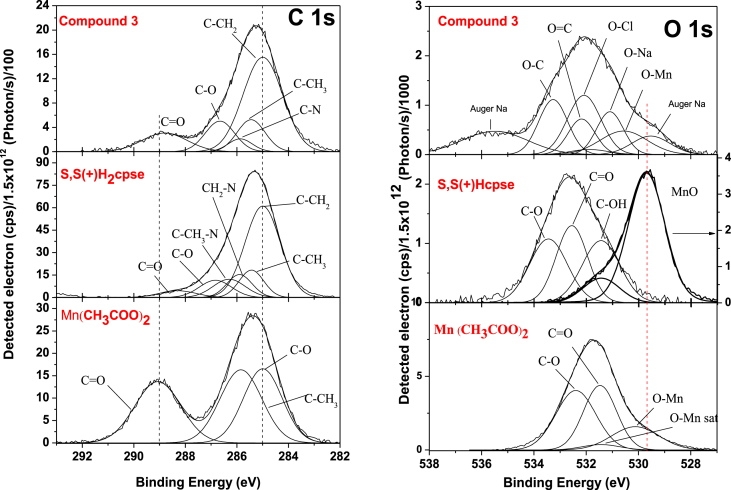
The high resolution XPS spectra of C 1s, and O 1s core level. XPS data of Mn(CH_3_COO)_2_, (S,S (+)H_2_cpse), and compound *[Mn*_*2*_*(S,S(* + *)Hcpse)*_*4*_*(NaClO*_*4*_*)*_*2*_*(NaOH)(CH*_*4*_*O)]*_*n*_*·[(C*_*2*_*H*_*6*_*O)*_*2*_*]*_*n*_*·[(CH*_*4*_*O)*_*2*_*]*_*n*_*.*

**Fig. 7:**
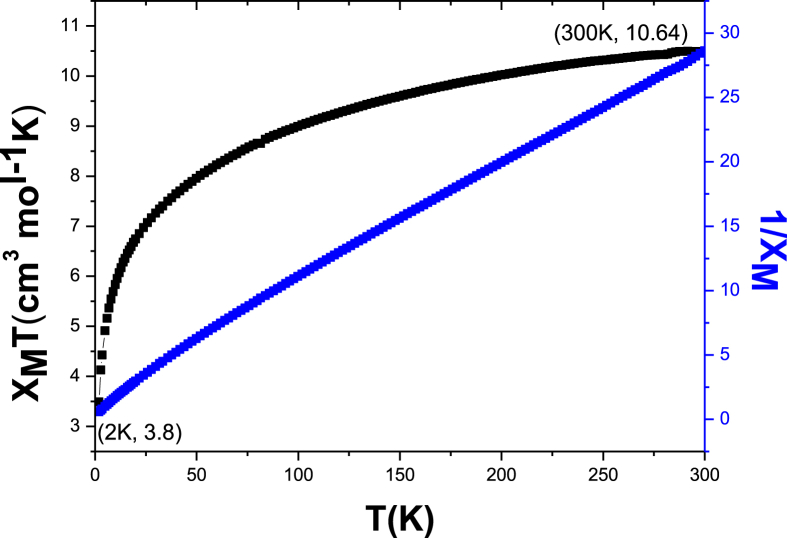
Temperature dependence of χ_M_T vs. T for a) [Mn_2_(S,S (+)Hcpse)_4_(NaClO_4_)_2_(NaOH)(CH_3_OH)]_n_·[(C_2_H_6_O)_2_]_n_·[(CH_4_O)_2_]_n_.

**Fig. 8:**
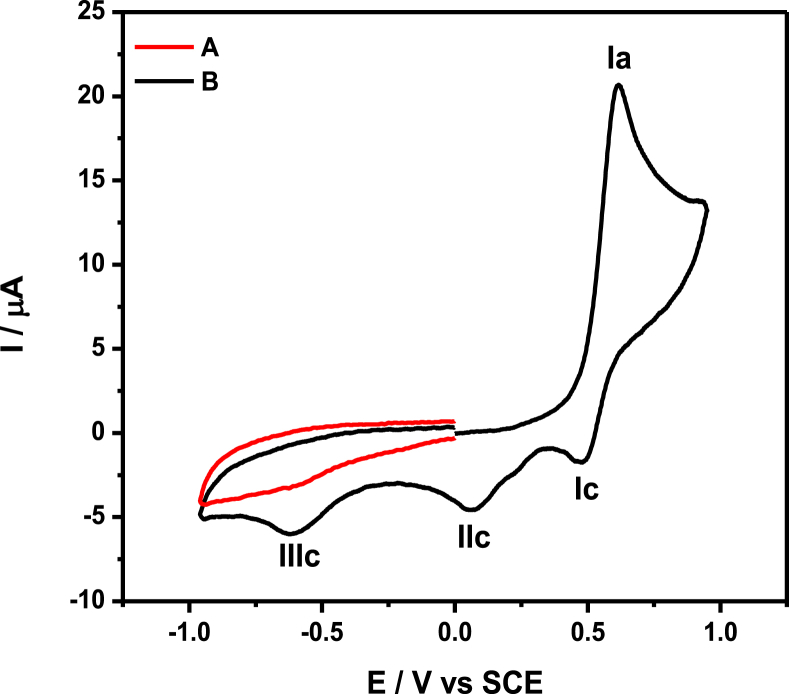
Cyclic voltammetry for **3**–1 mM, on glassy carbon electrode (ϕ = 3 mm), in acetonitrile containing *n*-Bu_4_NPF_6_ 0.1 M. a) positive direction starting scan, b) negative direction starting scan. Scan rate: 0.1 V s^−1^.

**Fig. 9:**
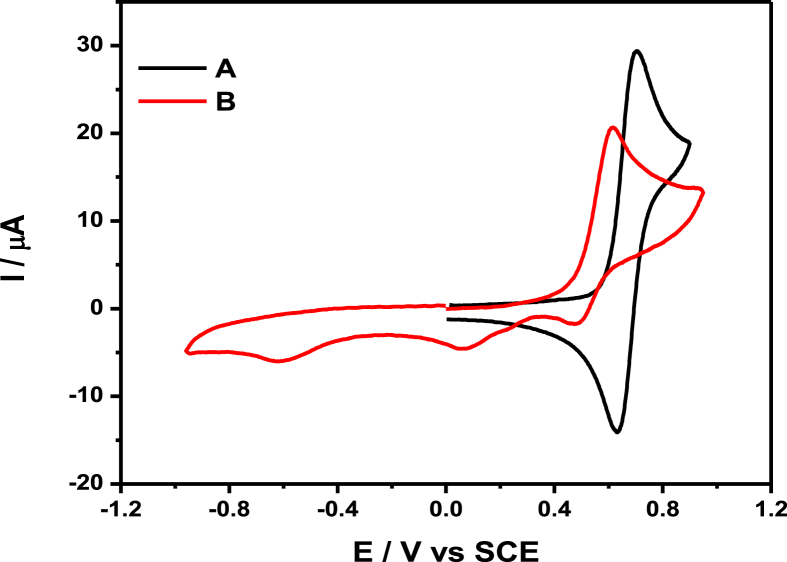
Comparison of the current due to the oxidation of ferrocenecarboxaldehyde 1 mM (A) with that for compound **3** ∼ 1mM (B), on glassy carbon electrode (ϕ = 3 mm), in acetonitrile containing *n*- Bu_4_NPF_6_ 0.1 M, at a scan rate of 0.1 Vs^−1^.

**Fig. 10:**
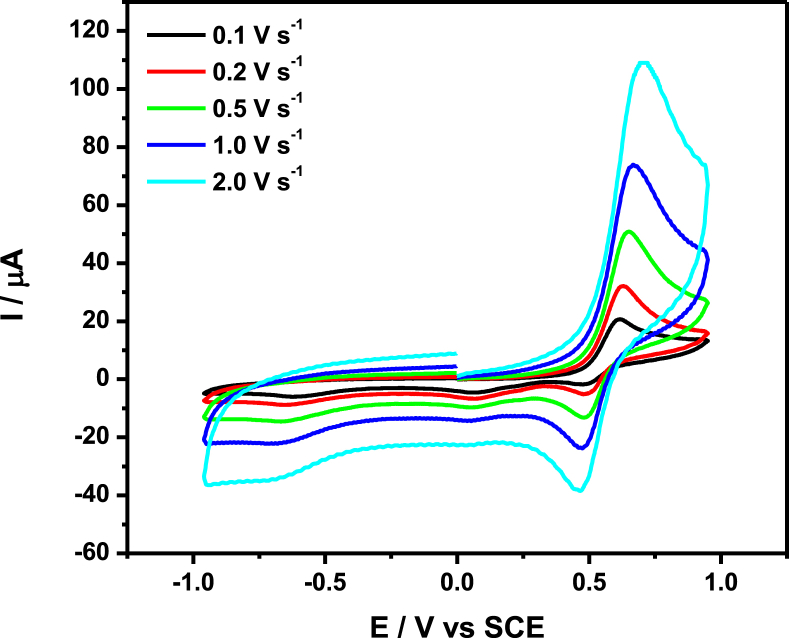
Cyclic voltammetry at different scan rates for **3**–1 mM, on glassy carbon electrode (ϕ = 3 mm), in acetonitrile containing *n*-Bu_4_NPF_6_ 0.1 M.

**Fig. 11:**
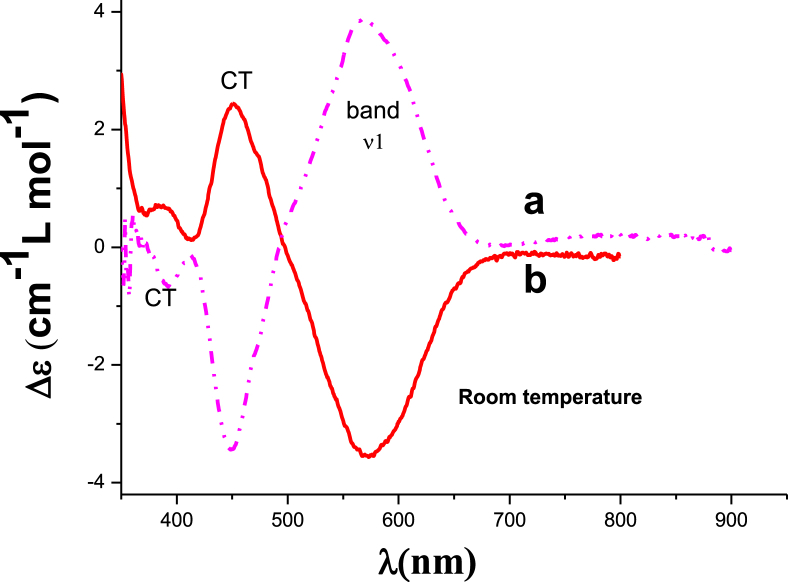
CD spectra for the manganese polymeric compounds. **a***. [Mn*_*2*_*(S,S(* + *)Hcpse)*_*4*_*(NaClO*_*4*_*)*_*2*_*(NaOH)(CH*_*3*_*OH)]*_*n*_*·[(C*_*2*_*H*_*6*_*O)*_*2*_*]*_*n*_*·[(CH*_*4*_*O)*_*2*_*]*_*n*_ and **b**. *[Mn*_*2*_*(R,R(−)Hcpse)*_*4*_*(NaClO*_*4*_*)*_*2*_*(NaOH)(CH*_*3*_*OH)]*_*n*_*·[(C*_*2*_*H*_*6*_*O)*_*2*_*]*_*n*_*·[(CH*_*4*_*O)*_*2*_*]*_*n*_*.*

**Fig. 12:**
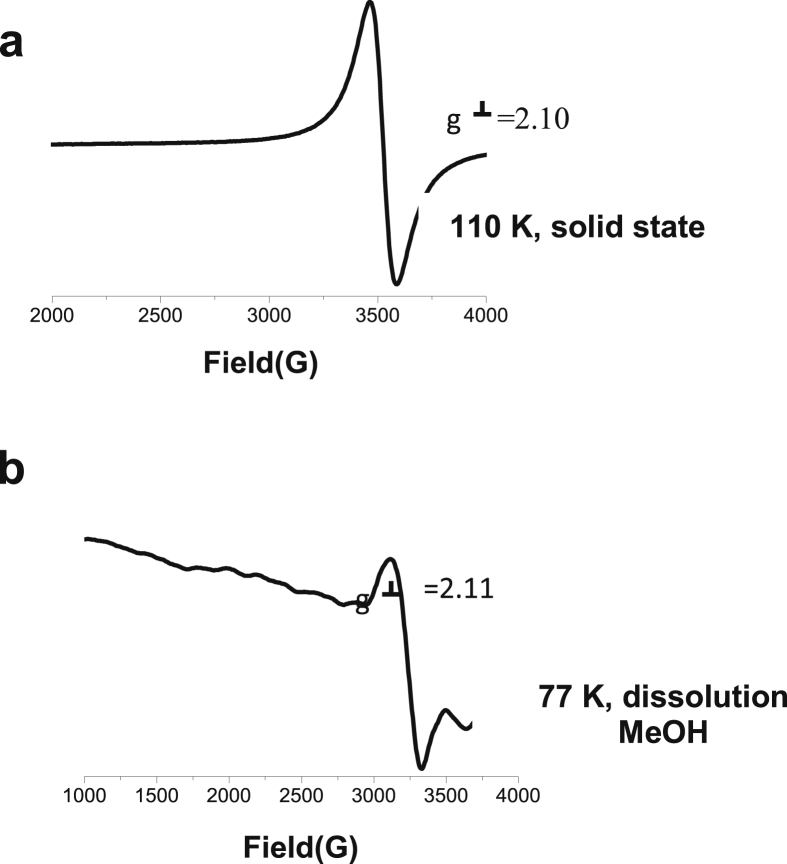
X-band spectra of *[Mn2(S,S(* + *)Hcpse)4(NaClO4)2(NaOH)(CH3OH)]n·[(C2H6O)2]n·[(CH4O)2]n***a**. 110 K, solid state; **b**. 77 K, dissolution, 2.03mM.

The data files of [Mn_2_(*S,S(*+)Hcpse)_4_(NaClO_4_)_2_(NaOH)(CH_4_O)]_n_·[(C_2_H_6_O)_2_]_n_·[(CH_4_O)_2_]_n_ and its respective isomer showed that in solid state the asymmetric unit is composed by a dinuclear manganese (II) entity, Table 1, Table 2, Table 3; likewise Hydrogen Bonds (°) and distances (Å) in Table 4 and Fig. 1, Fig. 2, Fig. 3. These structures showed similar coordination to metal center, as in metalloenzimes catalases. This was confirmed by X-ray crystal analysis, Fig. 4; spectra XPS, Fig. 5 and Fig. 6; and their antiferromagnetic properties, Fig. 7. Furthemore, in solution the electrochemical characterization indicated that the oxidation state for metal ions involved a slow electron transfer step followed by chemical reaction, which most probably corresponds to a bond breaking step, Fig. 8, Fig. 9, Fig. 10. Likewise, the presence of only a one-electron oxidation peak implies that the manganese possesses a single oxidation state in this complex, with dissociation of the dinuclear compound into a monomer, where the manganese ions presented oxidation (III) as valence state, in agreement with the CD, Fig. 11; and EPR measurements, Fig. 12; [[1], [2], [3], [4], [5], [6], [7]].

**Table 1:** Crystal data and structure refinement for [Mn_2_(*S,S(*+)Hcpse)_4_(NaClO_4_)_2_(NaOH)(CH_4_O)]_n_·[(C_2_H_6_O)_2_]_n_·[(CH_4_O)_2_]_n_.

Chemical formula (Sum)	C_51_H_79_Cl_2_Mn_2_N_4_Na_3_O_25_
Formula weight (Sum) (g mol^−1^)	1397.93
Crystal colour	dark purple
Crystal system	Monoclinic
Space group	C2
Unit cell dimensions	
a (Å)	21.5749 (8)
b (Å)	18.8865 (5)
c (Å)	17.6692 (6)
α (°)	90.0
β (°)	113.624 (4)
γ (°)	90.0
V (Å^3^)	6589.4 (4)
Z	4
^D^calc ^(g/cm3)^	1.409
F (000)	2920
Temp (K)	130
θ range (°)	3.3855–29.4984
Index range	−27 ≤ *h* ≤ 24
	−25 ≤ *k* ≤ 24
	*−24* ≤ *l* ≤ *21*
Reflections measured	27019
Independent reflections	15140
Reflections *[I > 2σ(I)]*	9048
R_int_	0.0660^a^
R	0.0621
R_w_	0.1363
S	0.928

a Rint=∑|Fo2−〈Fo2〉|/∑Fo2,R1=∑||Fo|−|Fc||/∑|Fo|,wR2=[∑w(Fo2−Fc2)/∑w(Fo2)2]12

**Table 2:** Selected bond distances (Å) of the *[Mn*_2_*(S,S(* + *)Hcpse)*_*4*_*(NaClO*_*4*_*)*_*2*_*(NaOH)(CH*_*4*_*O)]*_*n*_*[(C*_*2*_*H*_*6*_*O)*_*2*_*]*_*n*_*·[(CH*_*4*_*O)*_*2*_*]*_*n*_.

Mn1–O1	1.868 (4)
Mn1–O2	1.848 (5)
Mn1–O5	2.127 (5)
Mn1–O6	2.113 (4)
Mn1–N1	2.159 (5)
Mn1–N2	2.163 (6)
Mn2–O3	1.863 (4)
Mn2–O4	1.862 (5)
Mn2–O7	2.126 (4)
Mn2–O8	2.065 (5)
Mn2–N3	2.168 (5)
Mn2–N4	2.206 (5)
Mn1–Na3	3.384 (3)
Mn2–Na2^I^	3.611 (3)
Na1–Na2	3.339 (4)
Na2–Na3	3.581 (4)
Na3–Na4	3.606 (3)
Na1–O10	2.361 (4)
Na1–O12	2.469 (6)
Na1–O13	2.464 (9)
Na2–O7	2.737 (6)
Na2–O8	2.330 (5)
Na2–O10	2.340 (5)
Na2–O12	2.355 (6)
Na2–O21	2.341 (6)
Na2–O22	2.454 (6)
Na3–O5	2.343 (6)
Na3–O6	2.360 (5)
Na3–O7	3.048 (6)
Na3–O17	2.397 (10)
Na3–O11	2.271 (7)
Na3–O21	2.325 (7)
Na4–O9	2.395 (7)
Na4–O17	2.927 (15)
Na4–O11^I^	2.319 (5)
Na4–O11^II^	2.319 (5)
Na4–O9^III^	2.395 (7)
Na4–O17^III^	2.927 (15)

**Table 3:** Selected angles (°) for *[Mn*_*2*_*(S,S(* + *)Hcpse)*_*4*_*(NaClO*_*4*_*)*_*2*_*(NaOH)(CH*_*4*_*O)]*_*n*_*[(C*_*2*_*H*_*6*_*O)*_*2*_*]*_*n*_*·[(CH*_*4*_*O)*_*2*_*]*_*n*_ compound.

O2–Mn1–O1	172.3 (2)
O2–Mn1–O6	96.5 (2)
O1–Mn1–O6	90.4 (2)
O2–Mn1–O5	88.0 (2)
O1–Mn1–O5	96.9 (2)
O6–Mn1–O5	79.6 (2)
O2–Mn1–N1	92.0 (2)
O1–Mn1–N1	83.2 (2)
O6–Mn1–N1	154.7 (2)
O5–Mn1–N1	76.9 (2)
O2–Mn1–N2	83.2 (2)
O1–Mn1–N2	94.8 (2)
O6–Mn1–N2	77.4 (2)
O5–Mn1–N2	154.2 (2)
N1–Mn1–N2	127.4 (2)
O4–Mn2–O3	170.1 (2)
O4–Mn2–O8	93.0 (2)
O3–Mn2–O8	95.9 (2)
O4–Mn2–O7	94.4 (2)
O3–Mn2–O7	91.6 (2)
O8–Mn2–O7	79.6 (2)
O4–Mn2–N3	89.9 (2)
O3–Mn2–N3	83.3 (2)
O8–Mn2–N3	159.6 (2)
O7–Mn2–N3	80.1 (2)
O4–Mn2–N4	82.9 (2)
O3–Mn2–N4	94.4 (2)
O8–Mn2–N4	79.0 (2)
O7–Mn2–N4	158.2 (2)
N3–Mn2–N4	121.4 (2)
O10^I^–Na1–O10	179.4 (3)
O10^I^–Na1–O13	81.0 (3)
O10–Na1–O13	98.5 (3)
O10^I^–Na1–O13^I^	98.5 (3)
O10–Na1–O13^I^	81.0 (3)
O13–Na1–O13^I^	74.3 (6)
O10^I^–Na1–O12	103.3 (2)
O10–Na1–O12	77.2 (2)
O13–Na1–O12	105.2 (3)
O13^I^–Na1–O12	157.9 (3)
O10^I^–Na1–O12^I^	77.2 (2)
O10–Na1–O12^I^	103.3 (2)
O13–Na1–O12^I^	157.9 (3)
O13^I^–Na1–O12^I^	105.2 (3)
O12–Na1–O12^I^	83.6 (3)
O8^I^–Na2–O10	89.3 (2)
O8^I^–Na2–O21	152.1 (2)
O10–Na2–O21	93.1 (2)
O8^I^–Na2–O12	105.6 (2)
O10–Na2–O12	79.9 (2)
O21–Na2–O12	102.1 (2)
O8^I^–Na2–O22	78.0 (2)
O10–Na2–O22	157.6 (2)
O21–Na2–O22	106.7 (2)
O12–Na2–O22	85.8 (2)
O8^I^–Na2–O7^I^	63.3 (2)
O10–Na2–O7^I^	94.6 (2)
O21–Na2–O7^I^	88.8 (2)
O12–Na2–O7^I^	168.0 (2)
O22–Na2–O7^I^	96.0 (2)
O11^I^–Na3–O21	123.3 (2)
O11^I^–Na3–O5	88.9 (2)
O21–Na3–O5	146.5 (2)
O11^I^–Na3–O6	105.5 (2)
O21–Na3–O6	90.1 (2)
O5–Na3–O6	70.5 (2)
O11^I^–Na3–O17	82.3 (4)
O21–Na3–O17	109.5 (3)
O5–Na3–O17	81.5 (3)
O6–Na3–O17	150.5 (3)
O11^I^–Na4–O11^II^	162.9 (4)
O11^I^–Na4–O9^III^	81.5 (2)
O11^II^–Na4–O9^III^	112.4 (2)
O11^I^–Na4–O9	112.4 (2)
O11^II^–Na4–O9	81.5 (2)
O9^III^–Na4–O9	77.2 (4)
O11^I^–Na4–O17^III^	105.4 (2)
O11^II^–Na4–O17^III^	70.7 (2)
O9^III^–Na4–O17^III^	74.4 (3)
O9–Na4–O17^III^	127.9 (3)
O11^I^–Na4–O17	70.7 (2)
O11^II^–Na4–O17	105.4 (2)
O9^III^–Na4–O17	127.9 (3)
O9–Na4–O17	74.3 (3)
O17^III^–Na4–O17	154.5 (5)

Symmetry transformations used to generate equivalent atoms: ^I^ -x+1,y,-z+1, ^II^ x,y,z-1, ^III^ -x+1,y,-z, ^IV^ x,y,z+1, ^V^ -x+1/2,y+1/2,-z, ^VI^ -x+1/2,y-1/2,-z+1.

**Table 4:** Hydrogen Bonds (°) and distances (Å) of *[Mn*_2_*(S,S(* + *)Hcpse)*_4_*(NaClO*_4_*)*_2_*(NaOH)(CH*_*4*_*O)]*_*n*_*[(C*_2_*H*_6_*O)*_2_*]*_*n*_*·[(CH*_*4*_*O)*_*2*_*]*_*n*_.

D-H … A	d (D-H) (Å)	d `Å)	<(DHA) (°)
O1–H1A … O23		1.78	166.3
O3–H3 … O22^I^		1.84	178.5
O21–H21 … O20	0.85	2.34	122.7
O21–H21 … O22C	0.85	2.14	139.6
O23–H23C … O21	0.84	2.26	127.6
O24–H24C … O7^I^	0.85	2.66	179.4
O25–H25C … O24^I^	0.85	1.97	179.6
O25–H25D … O10^I^	0.85	1.89	179.6
C6–H6 … O15^I^		1.00	136.1
C17–H17C … O19^II^	0.98	2.65	167.4
C19–H19B … O14^III^	0.98	2.61	164.7
C26–H26 … O18^II^	0.98	2.54	165.7
C45–H45B … O20^II^	2.46	3.42	166.1
C37–H37 … Cg1^III^	0.98	2.84	145
C48–H48A … Cg2^III^	0.98	2.83	128

Symmetry transformations used to generate equivalent atoms: ^I^-x+1,y,-z+1, ^II^ -x+1/2,y+1/2,-z, ^III^ - x+1/2,y-1/2,-z+1. Cg1: C21→C26; Cg2: C27→C32.

X-ray diffraction data were measured using an Agilent Xcalibur Atlas Gemini diffractometer using graphite-monochromated Mo-Kα radiation and ω scans at 130 K. Analytical numeric absorption correction using a multifaceted crystal was applied, a summary for data collection and refinements is given in Table 1. The structure was solved using direct methods, using SHELXS- 2012 and the refinement (based on F2 of all data) was performed by full-matrix least-squares techniques with SHELXL-2014.24. The perchlorate anions, two phenyl rings (C21→C26 and C27→C32), coordinated and un-coordinated solvent molecules, exhibit orientational and/or statistical disorder which was modeled (mainly) over two positions. All disordered atoms (except the solvent methanol molecule C49–O22, which turn to be NPD and was refined isotopically) were refined with restraints (SIMU and DELU) on anisotropic displacement parameters and the bond geometries of the disordered groups were constrained or restrained to be similar with the AFIX or SADI commands of SHELXL. In addition, the contribution of a smeared un-coordinated solvent was “squeezed” using the PLATON program. IR spectra in the range 4000–400 cm^−1^ were recorded on a Nicolet FT-IR 740 spectrophotometer using KBr pellets. Electronic spectra (diffuse reflectance) were measured on a Cary 5E Uv–Vis–NIR spectrophometer in the range 250–2500 nm (40000 - 5000 cm^−1^). Elemental analyses were performed on a Fisons EA 1108 elemental analyser. Circular dichroism measurements were performed on a JASCO J815 spectrophotometer. Magnetic susceptibility measurements were carried out with a pendulum-type magnetometer (MANICS DSM8) equipped with an Oxford CF 1200 S helium continuous-flow cryostat working in the temperature range 300–400 K, in a magnetic field of 3 Oe. Powder diffraction was recorded on SIEMENS D500, graphite-monochromated Cu-Kα (λ = 1.5406 Å) radiation at 293 K, CT: 0.6 s, SS: 0.020 dg, WL = 1.5406 Å. The chemical analysis was obtained using X-ray photoelectron spectroscopy (XPS). This was performed using a VG Microtech ESCA2000 Multilab UHV system, with an Al K_α_ x-ray source (hν = 1486.6 eV), and a CLAM4 MCD analyser. XPS spectrum was obtained at 55° from the normal surface in the constant pass analyser energy mode (CAE), E_0_ = 50 and 20 eV for survey and high resolution narrow scan. Peak positions were referenced to the background silver 3d_5/2_ core level at 368.20 eV, having a FWHM of 1.00 eV, Au 4f_7/2_ in 84.00 eV and C 1s hydrocarbon groups in 285.00 eV central peak position. The XPS spectra were fitted with the program SDP v 4.1. Electrochemical experiments were carried out in a conventional three-electrode cell, with a glassy carbon disc (ϕ = 3 mm), a platinum mesh and a saturated calomel electrode (SCE) as working, auxiliary and reference electrodes, respectively. The supporting electrolyte was *n*-tetrabutylammonium hexafluorophosphate (*n*-Bu_4_NPF_6_) 0.1 M.

## 2 Experimental design, materials, and methods

### 2.1 Materials

The metal salt Mn(CH_3_COO)_2_·4H_2_O, NaClO_4_, NaOH, and methanol (J.T. Baker) were used without further purification. The synthesis of the ligands *N*-[2-hydroxy-1(*R*)-methyl-2(*R*)-phenylethyl]-*N*-methylglycine *(R,R(−)*H_2_cpse and *N*-[2-hydroxy-1(*S*)-methyl-2(*S*)-phenylethyl]-*N*-methylglycine (*S*,*S* (+)H_2_cpse) was carried out as previously described; [8].

### 2.2 Synthesis

#### 2.2.1 Synthesis of compound [Mn_2_(S,S(−)Hcpse)_4_(NaClO_4_)_2_(NaOH)(CH_4_O)]_n_·[(C_2_H_6_O)_2_]_n_·[(CH_4_O)_2_]_n_

S,S (+)H_2_cpse (479 mg, 2.14 mmol) in methanol (15 mL) was mixed with Mn(CH_3_COO)_2_·4H_2_O (265 mg, 0.108 mmol) and stirred during 15 minutes. Then, NaClO_4_ (13 mg, 0.109 mmol) was added to the mixture at basic pH and stirred again during 15 minutes. After two weeks brown needles, suitable for X-ray diffraction analysis, were obtained (Yield 84% (261 mg)). The compound showed to be hygroscopic. Calculated analysed (Anal. Calcd) for Mn_2_C_54_H_91_Cl_2_N_4_Na_3_O_28_: C%, 42.90; H%, 6.21; N%, 3.71. Exp.: C%, 43.15%; H% 5.77; N% 3.84. IR (KBrν/cm^−1^): 1569 (ν_as_ COO^−^) and 1443 (ν_s_COO^−^), Δν_(as-s)_ = 126 cm-1. MS: *m*/*z* [M]+, 99(87), 305(57), 146(38), 575(32), 875(30), 1179(13), 1098(12).

#### 2.2.2 Synthesis of compound [Mn_2_(R,R(−)Hcpse)_4_(NaClO_4_)_2_(NaOH)(CH_4_O)]_n_·[(C_2_H_6_O)_2_]_n_ [(CH_4_O)_2_]_n_

To a solution of R,R (−)H_2_cpse (481 mg, 2.16 mmol) in methanol (15 mL), Mn(CH_3_COO)_2_·4H_2_O (269 mg, 1.109 mmol) was added. The mixture was stirred for 15 minutes. After two weeks there was obtained a brown microcrystalline compound, not suitable for monocrystal X-ray diffraction analysis. Yield 87% (283 mg). The compound showed to be hygroscopic. Anal. Calcd for Mn_2_C_54·_H_89_C_l2_N_4_Na_3_O_27_: C%, 43.42; H%, 6.14; N%, 3.75. Exp.: C%, 43.85%; H%,5.93; N%, 3.75. IR (KBrν/cm^−1^): 1569 (ν_as_ COO^−^) and 1443 (ν_s_ COO^−^) Δν_(as-s)_ = 126 cm-1. These methodology has been used by Barba- Behrens and et-al for tridentate ligands derivate of *N*- Acetyl pseudoephedrine, [[8], [9], [10]].
